# Serum bone sialoprotein as a marker of tumour burden and neoplastic bone involvement and as a prognostic factor in multiple myeloma

**DOI:** 10.1054/bjoc.2000.1614

**Published:** 2001-02

**Authors:** H W Woitge, M Pecherstorfer, E Horn, A-V Keck, I J Diel, P Bayer, H Ludwig, R Ziegler, M J Seibel

**Affiliations:** Department of Medicine I, University of Heidelberg, Heidelberg, D-69115, Germany; Department of Gynecology & Obstetrics, University of Heidelberg, Heidelberg, D-69115, Germany; First Department of Medicine, Wilhelminenspital, Vienna, A-1171, Austria

**Keywords:** bone sialoprotein, multiple myeloma, tumour burden, neoplastic bone involvement, prognosis

## Abstract

To test the potential of immunoreactive BSP, a non-collagenous bone matrix component, as a clinical guide in patients with plasma cell dyscrasias, serum BSP concentrations were measured in 62 patients with newly diagnosed multiple myeloma (MM) followed over a period of 4 years, in 46 patients with monoclonal gammopathy of undetermined significance (MGUS), in 71 patients with untreated benign vertebral osteoporosis (OPO), and in 139 healthy adults. Results were compared with clinical and laboratory data, including serum osteocalcin (OC), and urinary pyridinoline (PYD) and deoxypyridinoline (DPD) as markers of bone turnover. In MM, serum BSP, and urinary PYD and DPD were higher than in healthy controls and in MGUS or OPO (*P*< 0.001). BSP levels correlated with the bone marrow plasma cell content (r = 0.40, *P*< 0.001), and serum β_2_-microglobulin (r = 0.31, P < 0.01). The differentiation of MM from healthy controls and from MGUS or OPO was highest for BSP. After chemotherapy, BSP reflected the response to treatment and correlated with the change in monoclonal protein (r = 0.55, *P*< 0.001). MM patients with normal baseline BSP levels survived longer than patients with initially elevated BSP values (*P*< 0.001, logrank test). Only serum monoclonal protein and BSP were independent predictors of survival. We conclude that in MM, BSP levels are associated with skeletal involvement and tumour cell burden. The quantification of serum BSP may be a non-invasive method for the diagnosis and follow-up, and may improve the prognostic value of conventional staging in MM. © 2001 Cancer Research Campaign http://www.bjcancer.com


*British Journal of Cancer* (2001) **84**(3), 344–351
© 2001 Cancer Research Campaign
doi:10.1054/ bjoc.2000.1614, available online at http://www.idealibrary.com on IDEAL®

http://www.bjcancer.com

### Serum bone sialoprotein as a marker of tumour burden and neoplastic bone involvement and as a prognostic factor in multiple myeloma

**HW Woitge[1], M Pecherstorfer[3], E Horn[3], A-V Keck[3], IJ Diel[2], P Bayer[3], H Ludwig[3], R Ziegler[1] and MJ Seibel[1]**

[1]Department of Medicine I and [2]Department of Gynecology & Obstetrics, University of Heidelberg, D-69115 Heidelberg, Germany; and the [3]First Department of Medicine, Wilhelminenspital, A-1171 Vienna, Austria

**Summary** To test the potential of immunoreactive BSP, a non-collagenous bone matrix component, as a clinical guide in patients with plasma cell dyscrasias, serum BSP concentrations were measured in 62 patients with newly diagnosed multiple myeloma (MM) followed over a period of 4 years, in 46 patients with monoclonal gammopathy of undetermined significance (MGUS), in 71 patients with untreated benign vertebral osteoporosis (OPO), and in 139 healthy adults. Results were compared with clinical and laboratory data, including serum osteocalcin (OC), and urinary pyridinoline (PYD) and deoxypyridinoline (DPD) as markers of bone turnover. In MM, serum BSP, and urinary PYD and DPD were higher than in healthy controls and in MGUS or OPO ($P < 0.001$). BSP levels correlated with the bone marrow plasma cell content ($r = 0.40$, $P < 0.001$), and serum $\beta_2$-microglobulin ($r = 0.31$, $P < 0.01$). The differentiation of MM from healthy controls and from MGUS or OPO was highest for BSP. After chemotherapy, BSP reflected the response to treatment and correlated with the change in monoclonal protein ($r = 0.55$, $P < 0.001$). MM patients with normal baseline BSP levels survived longer than patients with initially elevated BSP values ($P < 0.001$, logrank test). Only serum monoclonal protein and BSP were independent predictors of survival. We conclude that in MM, BSP levels are associated with skeletal involvement and tumour cell burden. The quantification of serum BSP may be a non-invasive method for the diagnosis and follow-up, and may improve the prognostic value of conventional staging in MM. © 2001 Cancer Research Campaign http://www.bjcancer.com

**Keywords**: bone sialoprotein; multiple myeloma; tumour burden; neoplastic bone involvement; prognosis

Tumour-induced bone disease is the major cause of morbidity in patients with multiple myeloma (MM) (Salmon and Cassady, 1993). The changes range from osteoporosis-like bone loss to the development of osteolytic lesions and frank hypercalcaemia (Salmon and Cassady, 1993; Malpas, 1995; Coleman, 1997). The process leading to MM-induced bone disease is based on a complex interplay of tumour cells, of cells of the bone marrow microenvironment and of bone matrix proteins. The initial step during the development of MM-induced bone disease is the homing of the myeloma cells in the bone marrow (Teoh and Anderson, 1997), which involves the interactions between adhesion molecules expressed on the cell surface of the tumour cells (e.g., the β integrins) and their receptors on cellular and non-cellular bone marrow elements (Teoh and Anderson, 1997; Van Riet et al, 1998). Adhesion of MM cells to cells of the bone marrow microenvironment triggers the secretion of cytokines (e.g., interleukin-6) by MM-cells and bone marrow stromal cells, which in turn increases the expression of adhesion molecules (Uchiyama et al, 1993). Interleukin-6 not only stimulates myeloma cell growth, but, like other cytokines (e.g., tumour necrosis factor and interleukin-1), is also responsible for the recruitment, differentiation and activation of osteoclasts (Bataille et al, 1997). The increased activity of osteoclasts together with the progressive decrease in the activity of bone-forming cells subsequently leads to MM-induced bone destruction (Bataille et al, 1989).

*Received 8 May 2000*
*Revised 19 October 2000*
*Accepted 8 November 2000*

*Correspondence to:* MJ Seibel

The evaluation of the degree of bone involvement in MM and in monoclonal gammopathies of undetermined significance (MGUS, plasma cell dyscrasias which evolve into malignant lymphoplasmocytic diseases in approximately one third of cases (Bladé and Kyle, 1995) could be important for clinical guidance (Bataille et al, 1986), but is limited by the invasiveness of the presently available standard method, the quantitative bone histology. This problem has been partly overcome by the quantification of biochemical markers of bone turnover, such as osteocalcin as a marker of bone formation (Bataille et al, 1990) and the degradation products of type I collagen as indexes of bone resorption (Elomaa et al, 1992; Pecherstorfer et al, 1997). We have recently shown that serum levels of circulating bone sialoprotein (BSP) also reflect bone turnover (Woitge et al 1999; Seibel et al, 1996). BSP is a phosphorylated 70–80 kDa glycoprotein that accounts for 5–10% of the non-collagenous bone matrix (Fisher et al, 1983; Heinegard and Oldberg, 1989). The protein is a major synthetic product of active osteo- and odontoblasts and contains an Arg-Gly-Asp (RGD) sequence (Chen et al, 1991; Fujisawa et al, 1993; Shapiro et al, 1993). This sequence is recognized by various integrin receptors including the $\alpha r \beta_3$ vitronectin receptor (CD51/CD61) (Oldberg et al, 1988; Ross et al, 1993), which is expressed by osteoclasts (Ross et al, 1993). Thus, in the course of normal bone remodelling, BSP is likely to be involved in the adhesion of bone resorbing cells to the extracellular bone matrix (Helfrich et al, 1992). Although preferentially detected in the cells of mineralized tissues (Fisher et al, 1983; Chen et al, 1991; Shapiro et al, 1993), BSP has been found to be ectopically expressed in the cytoplasma and on the surface of myeloma cells (Bellahcène et al, 1996b) and of tumour cells of other malignancies (Bellahcène et al, 1994). In studies of breast cancer patients, BSP was found to be associated with the appearance of bone metastases, suggesting that BSP may be involved in the molecular mechanisms responsible for cancer cell osteotropism (Bellahcène et al, 1996a; Diel et al, 1999).

Employing a recently developed radioimmunoassay (Karmatschek et al, 1997), the present study was carried out to evaluate the diagnostic and prognostic importance of serum BSP levels in MM and in MGUS. Healthy adults and patients with benign vertebral osteoporosis were included as control groups.

### SUBJECTS AND METHODS

### Subjects

A total of 318 individuals was included in the study. In all subjects, a complete history and physical exam was taken at the time of sample collection. The following groups were studied (Table 1).

#### *Healthy adults*

The control group comprised of an ambulatory population of 139 subjects without any evidence of skeletal or non-skeletal disease. All women in this group were postmenopausal with a mean duration of menopause ( ISD) of 13.2 I 9.7 years (median: 11.5 years, range: 1–30 years). All subjects had radiographs of the lumbar and thoracic spine (Multiplanimat Siemens, Erlangen, Germany). Bone mineral density was determined in an anterior-posterior position at the lumbar spine by dual X-ray absorptiometry (QDR 1000, Hologic, Waltham, MA). Subjects with vertebral fractures, a bone mineral density of less than 2 SD of the age- and sex-matched mean, significant degenerative disease of the spine, or abnormal laboratory results, were excluded. None of the controls was taking any medication known to affect bone metabolism, including bisphosphonates, glucocorticoids, HRT, osteotropic vitamins or calcium supplements.

#### *Patients with multiple myeloma (MM)*

62 patients with MM were enrolled in the study. All patients were studied at diagnosis of MM. Prior to study entry, none of the patients had been treated with chemotherapy, radiotherapy, glucocorticoids, or bisphosphonates. The diagnosis of MM was based on i) the percentage of plasma cells in the bone marrow, ii) the presence of monoclonal protein in serum or urine, iii) the radiological evidence of bone involvement. In all subjects, plain radiographs were taken of the skull, spine, pelvis and of painful regions outside the axial skeleton. Bone marrow biopsies were performed by Yamshidi technique. In all patients, serum creatinine was less than 4 mg $dl^{-1}$. Patients were followed over 4 years, with a median observation time of 427 days (range: 1–1550 days).

Staging was performed according to Durie and Salmon (1975). At the time of diagnosis, 14 patients (23%) presented with stage I, 15 patients (24%) with stage II, and 33 patients (53%) with stage III disease. One patient with stage I, 2 patients with stage II, and 6 patients with stage III disease had serum creatinine levels of ≥2 mg $dl^{-1}$ (substage B). For further analyses, patients were subclassified according to bone disease: i) no bone disease; ii) osteoporosis-like bone loss with or without pathological fractures; iii) osteolysis with or without pathological fractures; and iv) hypercalcaemia.

A protocol including vincristin, melphalan, cyclophosphamide, and prednisolone was applied in the majority of patients, except for 3 cases with indolent stage I MM and one patient who refused cytostatic treatment. None of the patients achieved a complete remission, i.e. a complete disappearance of the paraprotein as detected by immunofixation. 21 patients had sequential measurements on bone turnover markers and were included in the analyses of therapeutic monitoring.

#### *Patients with monoclonal gammopathy of undetermined significance (MGUS)*

46 patients with MGUS were included in the present study. All patients underwent plain X-ray examination of the skull, spine and pelvis, and bone biopsies were performed in Yamshidi technique. For diagnosis of MGUS and differentation to MM, the criteria of Salmon and Cassady were applied (Salmon and Cassady, 1993). As shown earlier, bone resorption rates in patients with smoldering myeloma do not differ from those in patients fulfilling strict

**Table 1** Population characteristics at baseline (median (range))

| | Healthy adults | MM | MGUS | OPO |
|---|---|---|---|---|
| Sex (M/F)* | 69/70 | 26/36 | 20/26 | 23/48 |
| Age (years) | 64 (37–83) | 68 (28–90) | 66 (40–86) | 67 (40–92) |
| No bone disease* | – | 9 | 29 | – |
| O'po* | – | 34 | 16 | 71 |
| O'ly* | – | 40 | – | – |
| S-Cr (mg $dl^{-1}$) | 0.9 (0.6–1.3) | 1.0 (0.41–3.9)$^{b}$ | 0.95 (0.7–1.7)$^{e}$ | 0.9 (0.5–1.8)$^{f}$ |
| S-Ca (mM $l^{-1}$) | 2.4 (2.16–2.65) | 2.37 (1.99–3.52) | 2.35 (1.93–3.09) | 2.39 (2.12–2.67) |
| U-Ca (mM $mM^{-1}$ Cr) | 0.25 (0.02–1.29) | 0.45 (0.01–23.4)$^{a}$ | 0.35 (0.04–3.11) | 0.42 (0.04–1.30) |
| β2 (mg $ml^{-1}$) | 1.7 (1.2–3.3) | 4.15 (1.4–16.3)$^{c}$ | 1.9 (0.9–6.3)$^{f}$ | 1.6 (1.0–7.6)$^{f}$ |
| t-Prot (mg $dl^{-1}$) | 7.3 (6.6–8.2) | 9.2 (5.7–14.3)$^{a}$ | 7.6 (6.2–9.7) | 7.3 (6.2–8.3) |
| CRP (mg $l^{-1}$) | 5.0 (5.0–50) | 8.6 (5.0–100)$^{a}$ | 5.0 (5.0–157) | 5.0 (5.0–26)$^{f}$ |
| S-Fer (µg $l^{-1}$) | 112 (10–699) | 190 (10–2315)$^{b}$ | 95 (8–586)$^{e}$ | 97 (10–843)$^{d}$ |
| Hb (g $dl^{-1}$) | 13.4 (11.1–15.6) | 11.05 (5.1–16.5)$^{c}$ | 13.7 (10.9–16.5)$^{f}$ | 13.9 (11.1–17.2)$^{a,f}$ |
| MP (%) | n.d. | 36 (0–65.7) | 18.5 (8.3–39.5)$^{f}$ | n.d. |
| PCC (%) | n.d. | 40 (0–95) | 5 (0–20)$^{f}$ | n.d. |

*Number of patients. $^{a}P < 0.05$, $^{b}P < 0.01$, $^{c}P < 0.001$ vs. healthy adults. $^{d}P < 0.05$, $^{e}P < 0.01$, $^{f}P < 0.001$ vs. MM.
Abbreviations: MM, multiple myeloma; MGUS, monoclonal gammopathy of undetermined significance; OPO; primary vertebral osteoporosis; M, males; F, females; O'po, osteoporosis-like bone involvement; O'ly, osteolysis; S-Cr, serum creatinine; S-Ca, serum calcium; U-Ca, urinary calcium; β2, β2-microglobulin in serum; t-Prot, total protein; CRP, C-reactive protein; S-Fer, serum ferritin; Hb, haemoglobin; MP, monoclonal protein in serum (% of total serum protein); PCC, plasma cell content of the bone marrow; n.d., not detected.

*© 2001 Cancer Research Campaign*

diagnostic criteria for MGUS (Bataille et al, 1996). We therefore included 5 patients in the MGUS group who were diagnosed with smoldering myeloma. The plasma cell content in these patients was higher than in the MGUS patients (>10% and <30% of nucleated cells), but none of them presented with anaemia, renal failure, or osteolytic bone lesions.

### *Patients with vertebral osteoporosis (OPO)*

71 patients with newly diagnosed and untreated osteoporosis were included. Of the 48 women in this group, all were post-menopausal with a median duration of menopause of 18 years (range: 2–41 years). The diagnosis of OPO was based upon the presence of at least one vertebral fracture (wedge, compression, or biconcave) that was not attributable to adequate spinal trauma, plus a lumbar bone mineral density below 2.5 SD of the age- and sex-matched mean. None of the subjects in this group had a history of malignant disease, nor were there any signs of plasma cell dyscrasia or secondary osteoporosis. At the time of study enrollment, all patients had normal renal and hepatic function, and none was given any medication known to interfere with bone turnover, including bisphosphonates, glucocorticoids, hormone replacement therapy, osteotropic vitamins or calcium supplementation.

### Laboratory analyses

Blood and urine samples were obtained simultaneously between 8 and 11 a.m. with subjects having had their usual breakfast. Venous blood was collected in vacutainers without additive, allowed to clot for 30 to 45 minutes at room temperature, and centrifuged at 1000 ***g*** for 10 minutes. Serum aliquots were stored at −80˚C. Urine samples were spot urines. Urinary creatinine (Cr) was determined immediately after sample collection, and a second aliquot was stored at –30˚C until analysis.

Routine biochemistry was performed in all participants, including RBC, WBL, serum calcium, creatinine, γ-glutamyl transpeptidase, albumin, β2-microglobulin, and serum and urine electrophoresis. No dietary restrictions were applied.

### *Determination of bone sialoprotein in serum*

Serum immunoreactive bone sialoprotein (BSP) was quantified by a new radioimmunoassay (RIA) described elsewhere (Karmatschek et al, 1997). In brief, BSP was isolated from human bone by standard extraction procedures (Fisher et al, 1987) and final purification was achieved by wide pore reversed-phase HPLC using a Latek C4 150 × 4 mm 300 A column and 0.13% heptafluorobutyric acid as eluant. Antibodies against human BSP were then raised in chicken and purified by affinity chromatography. For the determination of BSP in serum, 100 μl of $^{125}$I-labelled bone sialoprotein (1.5 ng ml$^{-1}$) and 100 μl of the chicken-anti-human-BSP-antibody (1:200 dilution) were added to an equal volume of serum, control sample, or standard, respectively. This mixture was incubated for 24 h at 4˚C, after which 100 μl l of a donkey-anti-chicken-IgG (1:150 dilution) were added as a second antibody. Following another incubation step (2 h, 4˚C), the antibody-bound radioactivity was centrifuged for 10 minutes at 2000 ***g***, and the supernatant was discarded. The radioactive pellet was then washed with 250 μl of an aqueous buffer (60 g l$^{-1}$ polyethylene glycol, 9 g l$^{-1}$ NaCl) and centrifuged for 10 minutes, after which the supernatant was discarded. Radioactivity was measured in a Beckmann - γcounter for 1 min, and results were calculated by interpolation of the unknown samples with a point-to-point curve fitting equation of the standards. The coefficients of variation were 7.0% for intra- (*n* = 20) and 9.1% for interassay variability (*n* = 10). The lower detection limit in the present RIA was 0.7 ng ml$^{-1}$. Spiking of human samples with purified BSP resulted in a mean recovery of 99.4% (range: 92–108%).

### *Determination of urinary pyridinium crosslinks*

Total urinary pyridinoline (PYD) and deoxypyridinoline (DPD) were determined by HPLC after acid hydrolysis of the urine (Black et al, 1988). Following partition chromatography on a CF1 cellulose column, pyridinium crosslinks of samples and external standards were separated by reverse-phase ion-paired HPLC, and the eluting crosslink compounds were quantified by fluorometry. The overall reproducibility of this assay was 8–12% including the partitioning step. Values were expressed relative to urinary creatinine (Cr) levels.

### *Determination of serum osteocalcin*

Serum osteocalcin (OC) was measured by a commercial immunoassay (LUMItest® Osteocalcin, BRAHMS Diagnostica, Berlin, FRG). Coefficients of variation, as assessed in our laboratory, were 6% for intra- (*n* = 20) and 9% for inter-assay variability (*n* = 10).

### Statistical analyses

The SAS software package was used for statistical analysis. Descriptive values are presented as median and range unless otherwise stated. Z scores are expressed as standard deviation of the age- and sex-adjusted mean of healthy adults according to: z = (x - mean)/SD. Linear regression analysis was performed to assess the correlations between markers. Group differences were determined using Student's *t*-test for parametric and Wilcoxon's rank-sum test for non-parametric variables. Fisher's exact test was used to test for differences in the distribution of patients within categories. Sex specific receiver operating characteristic (ROC) analyses were used to examine the diagnostic validity of the various markers. The association of variables to survival was determined by applying the Cox proportional hazards model, first univariately followed by a stepwise multivariate regression analysis. Survival rates, measured from the date of first diagnosis to death or last follow-up, were estimated using Kaplan-Meier analyses, and the logrank test was applied for detection of differences in survival curves. All statistical tests were two-tailed and a probability level of less than 0.05 was considered statistically significant.

### RESULTS

### Comparison of healthy controls and patients with MM, MGUS, or OPO

Median levels of serum BSP and of urinary PYD and DPD were significantly higher in patients with MM, MGUS and OPO than in normal controls (Table 2). Compared to healthy controls, median serum OC levels were significantly elevated only in patients with MM and OPO. Patients with MM had significantly higher levels of BSP, PYD and DPD than patients with MGUS or OPO. No significant differences in serum levels of OC were found among the 3 groups of patients.

© 2001 Cancer Research Campaign

**Table 2** Biochemical markers of bone turnover in the disease groups (median (range))

| | Healthy adults | | MM | | MGUS | | OPO | |
|---|---|---|---|---|---|---|---|---|
| | | ULN | | > ULN | | > ULN | | > ULN |
| BSP (ng ml$^{-1}$) | 10.1 (3.4–27.4) | 20.9 | 27.6 (4.8–120)[c] | 73% | 18.6 (3.7–76.1)[c,f] | 48% | 15.8 (7.4–82.2)[c,f] | 27% |
| OC (ng ml$^{-1}$) | 16 (6.9–37.1) | 34.4 | 24 (6.1–134)[b] | 30% | 19.1 (4.5–46) | 4% | 22.6 (12.2–45)[a] | 10% |
| PYD (nM mM$^{-1}$ Cr) | 23.8 (9.9–79.7) | 50.6 | 50.7 (15–774)[c] | 50% | 27.6 (8.9–63.6)[a,e] | 11% | 29.8 (11.4–101)[b,e] | 13% |
| DPD (nM mM$^{-1}$ Cr) | 5.5 (2–18.7) | 12.0 | 13.6 (3.9–314)[c] | 57% | 6.1 (2.2–21.8)[a,e] | 14% | 7.8 (2.7–21.5)[b,e] | 13% |

[a]$P < 0.05$, [b]$P < 0.01$, [c]$P < 0.001$ vs. healthy adults. [d]$P < 0.05$, [e]$P < 0.01$, [f]$P < 0.001$ vs. MM.
Abbreviations: see Table 1. ULN, upper limit of normal (95% confidence interval); BSP, serum immunoreactive bone sialoprotein; OC, serum osteocalcin; PYD, urinary pyridinoline; DPD, urinary deoxypyridinolin.

73% of patients with MM had serum levels of BSP above the upper limit of the normal range as compared to 48% of MGUS patients and 27% of patients with OPO. For the other markers, the proportion of patients with abnormally high values ranged from 30% to 50% in MM, from 4% to 14% in MGUS and from 10% to 13% in OPO (Table 2).

All 3 patients who showed a transformation from MGUS into overt MM during follow-up (after 19, 20 and 31 months, respectively) presented with initially elevated serum levels of BSP (21.0 ng ml$^{-1}$, 21.6 ng ml$^{-1}$ and 50.7 ng ml$^{-1}$, respectively). In contrast, the levels of PYD, DPD and OC of these patients were within normal range at the time of the first diagnosis of MGUS.

### Differentiation of MM patients from healthy adults and patients with MGUS or OPO

With regard to the differentiation between healthy subjects and patients with MM, results of ROC analyses were similar for serum BSP and the urinary crosslinks, whereas a clearly discrepant curve was obtained for serum OC (Figure 1A). For serum BSP, the area under the curve (AUC) was 0.922, and for urinary PYD and DPD 0.895 and 0.882, respectively. At a chosen specificity of 80%, serum BSP had a sensitivity of 87.5%, and similar values were seen at a specificity of 95%. In contrast, serum OC was not useful in distinguishing between healthy individuals and tumour patients (AUC: 0.502). At a specificity of 80%, this marker had a sensitivity of less than 40%. A similar but less pronounced pattern of results was noted when patients with MM were compared to patients with MGUS (Figure 1B) or OPO (Figure 1C).

### Effect of the tumour stage and of the extent of bone disease on biochemical markers of bone metabolism

When MM patients were stratified according to the staging system of Durie and Salmon, patients with stage II and stage III disease had significantly higher serum levels of BSP than patients with stage I disease ($P < 0.01$) and healthy controls ($P < 0.001$). In patients with stage I disease, BSP levels were also elevated compared to healthy controls ($P < 0.001$). BSP levels in patients with stage II disease did not differ from those with stage III disease. Compared to healthy controls, urinary PYD and DPD were higher in MM patients with stage II ($P < 0.01$) and stage III disease ($P < 0.01$), but not stage I disease. Urinary DPD levels were significantly higher in patients with stage III disease than in patients with stage I disease ($P < 0.05$). Serum levels of OC did not differ among the 3 stages (Figure 2).

Stratification according to the extent of neoplastic bone involvement, as judged from plain radiographs and the measurement of serum calcium, revealed a similar pattern for serum BSP and the urinary crosslinks (Figure 3): Hypercalcaemic patients and normocalcaemic patients with overt osteolysis had significantly higher levels of all 3 markers than healthy adults ($P < 0.01$) and patients with MGUS ($P < 0.03$). Normocalcaemic MM patients with osteoporosis-like changes and MM patients without radiologically evident bone destruction had significantly higher values of serum BSP ($P < 0.01$) and urinary DPD ($P < 0.05$) compared to healthy adults, but no significant differences were detected when compared to patients with MGUS. Also, no significant differences were found between MM patients with osteoporosis-like bone loss and MM patients with lytic lesions for any of the applied markers of bone turnover (Figure 3).

Since the differentiation between benign and neoplastic osteoporosis-like changes is of particular clinical interest, biochemical markers of 11 MM patients with osteoporosis-like bone involvement without evidence for lytic lesions were compared with the corresponding marker levels of 11 age- and sex-matched patients with benign osteoporosis. Serum BSP and urinary PYD were significantly higher in the MM group ($P < 0.05$, respectively). No significant differences were found for serum OC and urinary DPD.

### Correlations of laboratory parameters in patients with MM

Serum concentrations of BSP correlated with serum creatinine ($r = 0.25$, $P < 0.05$), serum calcium ($r = 0.44$, $P < 0.001$), β2-microglobulin ($r = 0.31$, $P < 0.01$), serum ferritin ($r = 0.35$, $P < 0.01$), haemoglobin ($r = 0.26$, $P < 0.05$), and with the plasma cell infiltration in the bone marrow ($r = 0.40$, $P < 0.001$). Of the other markers of bone turnover, only DPD showed a weak correlation with the plasma cell infiltration in the bone marrow ($r = 0.19$, $P < 0.05$). No other significant correlations were found. Serum β2-microglobulin was positively associated with serum creatinine ($r = 0.42$, $P < 0.001$), serum calcium ($r = 0.28$, $P < 0.05$), and with the plasma cell infiltration in the bone marrow ($r = 0.50$, $P < 0.001$), and showed an inverse correlation with haemoglobin ($r = 0.43$, $P < 0.001$). Except for PYD and DPD, no significant correlations were observed among the different biochemical markers of bone turnover.

### Effect of chemotherapy on serum BSP in patients with MM

In 21 patients, markers of bone turnover were reanalysed following chemotherapy. In all 12 patients with a > 25% decrease in the monoclonal component, a reduction in serum BSP levels was found ($P < 0.001$ vs. baseline; average decrease of BSP:

*© 2001 Cancer Research Campaign*

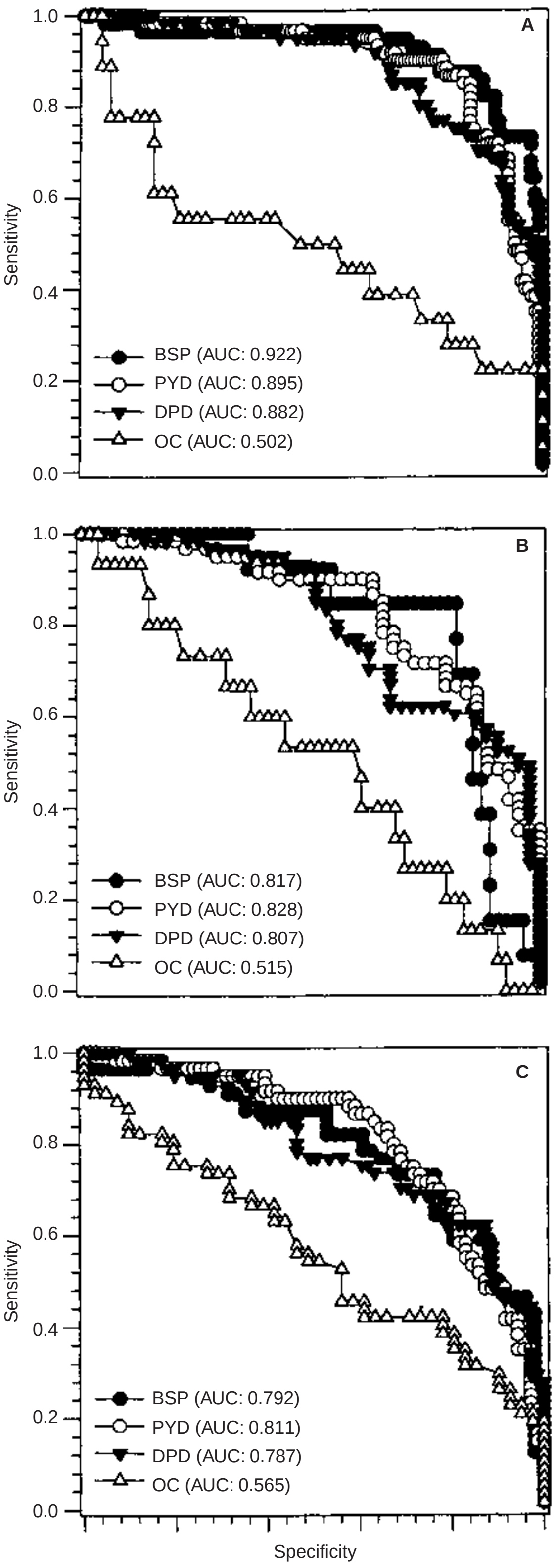

**Figure 1** Discriminative power of biochemical markers of bone turnover in patients with MM vs. healthy subjects, patients with MGUS, or OPO. Results of ROC analyses are given as the respective area under the curve (i.e. the mean sensitivity across the range of possible specificities). The differentiation between patients with MM and (**A**) healthy subjects, (**B**) MGUS, and (**C**) OPO is shown for each individual biochemical marker. Abbreviations: see Tables 1 and 2; AUC, area under the curve

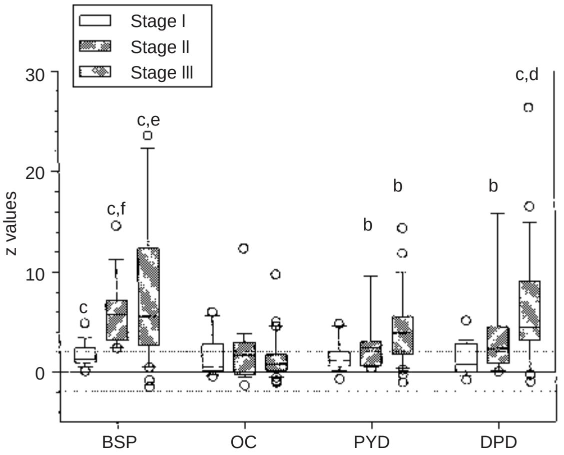

**Figure 2** Biochemical markers of bone turnover in patients with MM stratified according to the disease stage. Values are expressed as z scores (see Methods section). The full lines represent the mean, and the dotted lines represent ± 2 SD around the mean of healthy controls. [a]$P < 0.05$, [b]$P < 0.01$, [c]$P < 0.001$ vs. healthy controls. [d]$P < 0.05$, [e]$P < 0.01$, [f] $P < 0.001$ vs. stage I. Abbreviations: see Tables 1 and 2

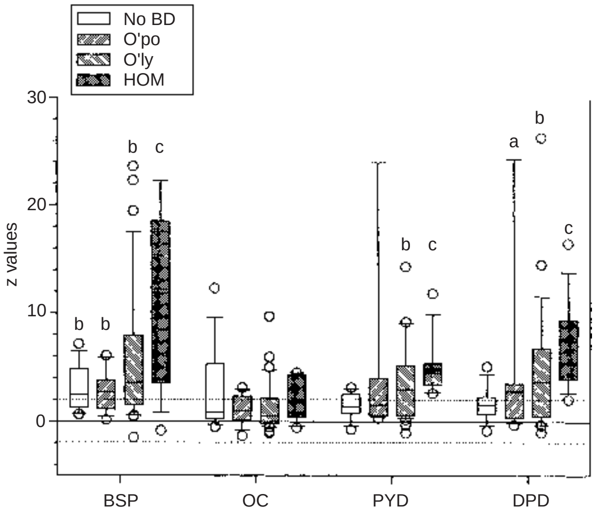

**Figure 3** Biochemical markers of bone turnover in patients with MM stratified according to the type of bone disease. Values are expressed as z scores (see Methods section). The full lines represent the mean, and the dotted lines represent ± 2 SD around the mean of healthy controls. [a]$P < 0.05$, [b]$P < 0.01$, [c]$P < 0.001$ vs. healthy controls. Abbreviations: see Tables 1 and 2; BD, bone disease; HOM, hypercalcaemia of malignancy

53%;) after a median observation period of 5 months (range: 1–10 months). In contrast, in patients without a significant response or an increase in the monoclonal protein, serum BSP levels did not change significantly after a median observation period of 6 months (range: 1–7 months) (Figure 4). The proportional change in serum BSP levels was significantly associated with the proportional change in monoclonal protein concentrations ($r = 0.55$, $P < 0.001$). Laying down a 50% reduction in BSP levels as response criteria, 8 of the 21 patients achieved a BSP response. All of these patients showed a > 25% decrease in the monoclonal component, thus, could be regarded as responders to therapy. In this group, the change in serum BSP levels was highly correlated with the propor-

© 2001 Cancer Research Campaign

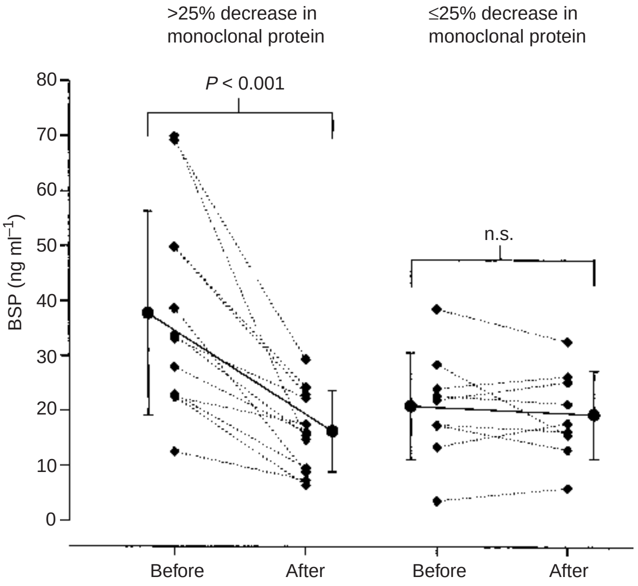

**Figure 4** Serum BSP before and after chemotherapy. 21 patients were analysed according to serum BSP levels in response to a VMCP regimen. The left panel shows the result for the responders (*n* = 12), i.e. > 25% decrease in the monoclonal protein, with a median observation period of 5 months (range: 1–10 months). The right panel shows the result for the non-responders (*n* = 9), i.e. ≤ 25% or increase in the monoclonal protein, with a median observation period of 6 months (range: 1–17 months). Small symbols and dotted lines represent individual values, large symbols (± SD) and full lines represent the mean of each group

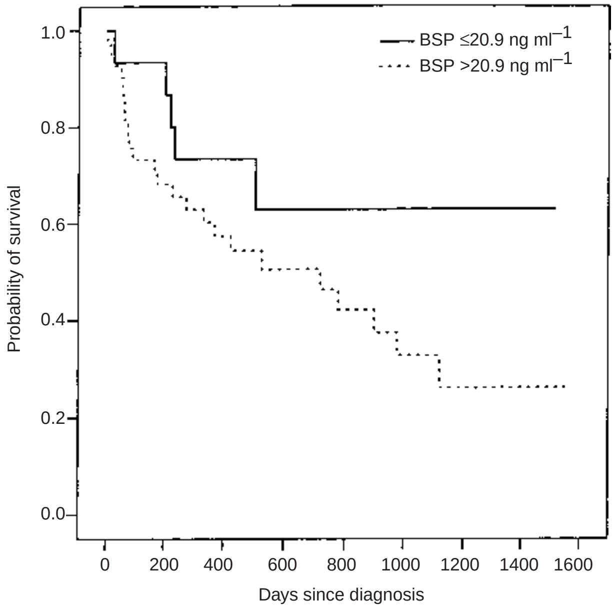

**Figure 5** Probability of survival according to serum BSP. Kaplan-Meier estimates are shown for the group of patients with serum BSP ≤20.9 ng $ml^{-1}$ (*n* = 15, full line) and for the group of patients with serum BSP > 20.9 ng $ml^{-1}$ (*n* = 41, dotted line). Differences in the survival probabilities from the time of diagnosis until death or latest follow-up were estimated using the log rank test. The median survival times of the two groups are significantly different ($P < 0.001$)

tional change in monoclonal protein concentrations (r = 0.76, $P < 0.001$). Another subanalysis included 10 of the 21 patients who had follow-up measurements after 6 ± 1 months. 6 of these patients were responders to therapy with a > 25% decrease in the monoclonal component and showed a decrease in BSP levels ranging from 28–58% (median: 51%). In contrast, the 4 non-responders in this subgroup showed a marginal decrease (<10%) or even an increase in serum BSP.

**Table 3** Disease characteristics by BSP levels in patients with MM (median (range))

| | **BSP ≤ 20.9 ng $ml^{-1}$ (I)** | **BSP > 20.9 ng $ml^{-1}$ (II)** |
|---|---|---|
| Sex (M/F)* | 6/9 | 20/27 |
| Age (y) | 70 (28–90) | 67 (39–87) |
| Paraproteinaemia | | |
| IgG | 80% | 78% |
| IgA | 20% | 15% |
| Others | 0% | 7% |
| S-Cr (mg $dl^{-1}$) | 1.1 (0.6–3.3) | 1.0 (0.41–3.9) |
| S-Ca (mM $l^{-1}$) | 2.28 (2.03–2.88) | 2.39 (1.99–3.52)$^{P = 0.077 \text{ vs. I}}$ |
| U-Ca (mM $mM^{-1}$ Cr) | 0.33 (0.07–1.02) | 0.51 (0.01–23.4)$^{P = 0.086 \text{ vs. I}}$ |
| β2 (mg $l^{-1}$) | 3.8 (1.6–13.4) | 4.4 (1.4–16.3) |
| t-Prot (mg $dl^{-1}$) | 10.1 (5.7–14.2) | 8.7 (6.0–14.3) |
| CRP (mg $dl^{-1}$) | 0.52 (0.5–10) | 0.73 (0.5–7.1) |
| S-Fer (µg $l^{-1}$) | 88 (30–1483) | 223 (10–2315) |
| Hb (g $dl^{-1}$) | 11.3 (7.9–13.8) | 11 (5.1–16.5) |
| MP (%) | 42.8 (17.5–64.7) | 35.6 (0–65.7) |
| PCC (%) | 15 (0–80) | 50 (0–95)$^{P = 0.062 \text{ vs. I}}$ |

*Number of patients. Abbreviations: see Table 1.

**Table 4** Univariate Cox Proportional Hazards Analysis*

| Variable | Coefficient | SE | $\chi^2$ | *P*= |
|---|---|---|---|---|
| MP | 0.0327 | 0.0121 | 7.534 | 0.0061 |
| β2 | 0.1174 | 0.0443 | 7.299 | 0.0069 |
| PCC | 0.0180 | 0.0069 | 7.208 | 0.0073 |
| BSP | 0.0135 | 0.0056 | 6.219 | 0.0126 |
| t-Prot | 0.1467 | 0.0768 | 3.720 | 0.0538 |
| Stage[a] | 0.4550 | 0.2470 | 3.521 | 0.0606 |

*Univariate survival analyses were performed for all variables listed in Tables 1 and 2. Only those variables (continuous, except [a]) are presented that reached *P* values of <0.1. Abbreviations: see Table 1.

### Serum BSP as a prognostic factor in patients with MM

The probability of survival was significantly greater in the 15 patients with initially normal serum BSP values (≤20.9 ng $ml^{-1}$) compared to the 41 patients with BSP values above the normal range ($P < 0.001$, logrank test) (Figure 5). When patients were stratified according to BSP values within or above the normal range, no statistically significant differences in other clinical and laboratory values were found (Table 3). Interestingly, in a subgroup of patients with baseline levels of BSP > 70 ng $ml^{-1}$ (*n* = 10), no patient survived the observation period (median survival time: 92 days).

Univariate analyses using the Cox proportional hazards model identified the monoclonal protein ($P = 0.0061$), β2-microglobulin ($P = 0.0069$), the plasma cell content of the bone marrow ($P = 0.0073$), and serum BSP ($P = 0.0126$) as prognostic parameters for shortened survival (Table 4). However, a stepwise multivariate regression analysis (including all variables with a *P* value of < 0.1

*© 2001 Cancer Research Campaign* 

**Table 5** Multivariate Cox Proportional Hazards Analysis*

| Variable | Coefficient | SE | $\chi^2$ | *P*= |
|---|---|---|---|---|
| BSP | 0.0133 | 0.0054 | 6.382 | 0.0115 |
| MP | 0.0937 | 0.0346 | 4.826 | 0.0280 |

*All variables listed in Table 4 were included in the stepwise multivariate regression analysis. Only those variables are presented that reached *P* values of <0.05. Abbreviations: see Table 1.

in the univariate analysis) revealed that only BSP and the monoclonal protein independently contributed to the prediction of survival (Table 5). Exclusion of 3 cases with indolent stage I MM from the analyses did not affect the results.

### DISCUSSION

In the present investigation, MM patients were found to have significantly higher serum BSP levels than did healthy adults and patients with MGUS or benign osteoporosis. Our earlier finding that MM patients present with increased levels of urinary pyridinium crosslinks, which up to now have been regarded as the most reliable biochemical markers of bone resorption, was confirmed (Pecherstorfer et al, 1997). In MGUS patients, the median BSP levels were also significantly elevated when compared to values in age-and sex-matched healthy adults (48% of MGUS patients had BSP levels above the upper limit of the normal range), but did not differ from levels in patients with benign osteoporosis (Table 1). Previously, Bataille et al showed an increased bone resorption rate (as assessed by quantitative bone histology) in 45% of MGUS patients at the time of diagnosis (Bataille et al, 1996). The authors also noted that the bone resorption rate at presentation was significantly higher in MGUS patients who subsequently showed a progression into overt MM than in MGUS patients with stable disease. In the present study, all 3 individuals with MGUS that later was found to have transformed into MM, revealed elevated BSP levels at the time of the first diagnosis of MGUS. Thus, BSP serum levels may serve as an early marker of malignant transformation in plasma cell dyscrasias initially classified as MGUS.

Serum BSP as well as urinary PYD and DPD were able to discriminate between patients with MM and healthy controls, as shown by ROC analysis. Of even greater clinical importance is the differentiation between tumour-induced and benign osteoporosis. In this regard, we found that serum BSP and urinary PYD were significantly higher in a subgroup of patients with MM-induced osteoporosis-like bone loss than in age- and sex-matched patients with benign osteoporosis. However, ROC analyses demonstrated that none of the applied biomarkers of bone turnover had enough discriminative power (AUC in ROC analysis > 0.9) to differentiate between MM and OPO. Therefore, it seems unlikely that a single non-invasive measurement of bone metabolism will be sufficient to discriminate between benign and malignant bone disease.

Compared to the urinary pyridinium crosslinks, serum BSP appears to be more sensitive in reflecting early interactions between myeloma cells and the bone resorbing cells. While in stage I myeloma patients PYD and DPD values did not differ from those in healthy adults, median BSP levels were found to be significantly higher (Figure 2). This finding could be explained by the fact that BSP is produced by bone forming cells. The number and activity of osteoblasts are increased in early stage MM (Bataille et al, 1991), but become progressively suppressed as the malignant process progresses (Bataille et al, 1989). Additional BSP may origin directly from MM cells (Bellahcène et al, 1996b). Thus, while the urinary pyridinium crosslinks only reflect the amount of collagen degradation during osteolysis, serum BSP levels may be influenced by the number and activity of osteoblasts and also by the number of MM cells and their BSP production rate. The latter assumption is supported by the significant correlations between serum BSP levels and two established markers of tumour load: the serum concentrations of β2-microglobulin and the plasma cell content of the bone marrow (Salmon and Cassady, 1993). A correlation with the tumour load could not be shown for the urinary pyridinium crosslinks.

Following chemotherapy, the significant correlation between changes in serum BSP concentrations and changes in the monoclonal protein further indicates that serum BSP levels reflect tumor burden. All patients with objective improvement (> 25% reduction in the monoclonal protein) under the VMCP regimen also showed a significant reduction in their BSP levels. No such changes were found for the pyridinium crosslinks (Pecherstorfer et al, 1997).

We here demonstrate that BSP serum levels at the time of first diagnosis are negatively associated with the survival time of MM patients. In a univariate analysis, the serum concentrations of the monoclonal protein, of β2-microglobulin and of BSP as well as the plasma cell content of the bone marrow were significant predictors of the survival time. However, in a subsequent multivariate analysis only serum BSP and the monoclonal protein were found to be independent prognostic indices (Table 5).

In breast and prostate cancer, the expression of BSP has already been shown to be highly predictive of neoplastic bone involvement (Bellachène et al, 1996a; Waltregny et al, 1998; Diel et al, 1999). In these cancers, as well as in myeloma, the interaction between tumour cells and osteoclasts seems to be pivotal for the growth and propagation of the neoplastic cell clone in the bone marrow and for the subsequent bone destruction (Mundy and Yoneda, 1998). BSP may play a central role in the relationship between MM cells and osteoclasts. Raynal et al demonstrated that BSP increases bone resorption in vitro in a dose-dependent manner (Raynal et al, 1996). Therefore, we hypothesize that following the homing of the MM cells to the bone marrow, the secretion of BSP by the neoplastic cells increases the bone resorption rate. The release of BSP and growth factors (e.g., transforming growth factor-β) and other proteins already present in the bone matrix in turn stimulate directly or indirectly (via stimulation of other cells of the bone marrow microenvironment) the proliferation of the tumour cells (Bataille et al, 1997; Mundy and Yoneda, 1998). It should also be noted that MM cells have been shown to express an integrin receptor, i.e. the vitronectin receptor, that binds to the RGD region of BSP (Van Riet and Van Camp, 1993), although, however, to a lesser extent than other adhesion molecules such as CD44, VLA-4 and VLA-5, syndecan-1 and NCAM (Van Riet et al, 1998).

In conclusion, serum BSP levels were significantly increased in patients with MM and were found to correlate with bone destruction, tumour mass and overall survival. The measurement of BSP serum levels is – in contrast to quantitative bone histology – a noninvasive procedure, which can easily be repeated as required. The quantification of immunoreactive BSP in serum also seems to overcome some of the limitation of urinary bone turnover markers, i.e. the greater technical and biological variability. Serum BSP may serve as a parameter for quantifying myeloma-induced bone

 *© 2001 Cancer Research Campaign*

turnover and may be used to estimate the tumour burden and to evaluate the effects of anti-myeloma and anti-resorptive (Woitge et al, 1999) therapy.

### ACKNOWLEDGEMENTS

We thank Antje von Schickfuss, Beatrice Auler, and Dr Christian Kissling for excellent technical assistant. The serum BSP assay was commercially available from Immundiagnostik (Bensheim, Germany).

### REFERENCES

Bataille R, Chappard D, Alexandre C and Sany J (1986) Importance of quantitative histology of bone changes in monoclonal gammopathy. *Br J Cancer* **53**: 805–810

Bataille R, Chappard D, Marcelli C, Dessauw PH, Baldet P, Alexandre C and Sany J (1989) Mechanism of bone destruction in multiple myeloma: the importance of an unbalanced process in determining the severity of lytic bone disease. *J Clin Oncol* **7**: 1909–1914

Bataille R, Delmas PD, Chappard D and Sany J (1990) Abnormal serum bone Gla protein levels in multiple myeloma: crucial role of bone formation and prognostic implications. *Cancer* **66**: 167–172

Bataille R, Chappard D, Marcelli C, Dessauw P, Baldet P, Sany J and Alexandre C (1991) Recruitment of new osteoblasts and osteoclasts is the earliest critical event in the pathogenesis of human multiple myeloma. *J Clin Invest* **88**: 62–66

Bataille R, Chappard D and Basle MF (1996) Quantifiable excess of bone resorption in monoclonal gammopathy is an early symptom of malignancy: a prospective study of 87 bone biopsies. *Blood* **87**: 4762–4769

Bataille R, Manolagas SC and Berenson JR (1997) Pathogenesis and management of bone lesions in multiple myeloma. *Hematol Oncol Clin North Am* **11**: 349–361

Bellahcène A, Merville MP and Castronovo V (1994) Expression of bone sialoprotein, a bone matrix protein, in human breast cancer. *Cancer Res* **54**: 2823–2826

Bellahcène A, Kroll M, Liebens F and Castronovo V (1996a) Bone sialoprotein expression in primary human breast cancer is associated with bone metastases development. *J Bone Miner Res* **11**: 665–670

Bellahcène A, van Riet I, Antoine N, van Camp B and Castronovo V (1996b) Expression of bone sialoprotein in myeloma cell lines. *Proc Annu Meet Am Assoc Cancer Res* **37**: 618 (abstr)

Black D, Duncan A and Robins SP (1988) Quantitative analysis of the pyridinium crosslinks of collagen in urine using ion-paired reversed-phase high-performance liquid chromatography. *Anal Biochem* **169**: 197–203

Bladé J and Kyle RA (1995) Monoclonal gammopathies of undetermined significance. In: *Myeloma. Biology and Management*, Malpas JS, Bergsagel DE, Kyle RA (eds) pp. 433. Oxford University Press: Oxford

Chen JK, Shapiro HS, Wrana JL, Reimers S, Heersche JN and Sodek J (1991) Localization of bone sialoprotein (BSP) expression to sites of mineralized tissue formation in fetal rat tissues by in situ hybridization. *Matrix* **11**: 133–143

Coleman RE (1997) Skeletal complications of malignancy. *Cancer* **80**: 1588–1594

Diel IJ, Solomayer EF, Seibel M, Pfeilschifter J, Gollan C, Conradi R, Meisenbacher H, Naser W, Hoyle N and Bastert G (1999) Serum bone sialoprotein in patients with primary breast cancer is a prognostic marker for subsequent bone metastasis. *Clin Cancer Res* **5**: 3914–3919

Durie BGM and Salmon SE (1975) A clinical staging system for multiple myeloma. Correlation of measured myeloma cell mass with presenting clinical features, response to treatment and survival. *Cancer* **36**: 842–854

Elomaa I, Virkkunen P, Risteli L and Risteli J (1992) Serum concentration of the cross-linked carboxyterminal telopeptide of type I collagen (ICTP) is a useful prognostic indicator in multiple myeloma. *Br J Cancer* **66**: 337–341

Fisher LW, Whitson SW, Avioli LW and Termine JD (1983) Matrix sialoprotein of developing bone. *J Biol Chem* **258**: 12723–12727

Fisher LW, Hawkins GR, Tuross N and Termine JD (1987) Purification and partial characterization of small proteoglycans I and II, bone sialoproteins I and II, and osteonectin from the mineral compartment of developing human bone. *J Biol Chem* **262**: 9702–9708

Fujisawa R, Butler WT, Brunn JC, Zhou HY and Kuboki Y (1993) Differences in composition of cell-attachment sialoproteins between dentin and bone. *J Dent Res* **72**: 1222–1226

Heinegard D and Oldberg A (1989) Structure and biology of cartilage and bone matrix noncollageneous macromolecules. *FASEB J* **3**: 2042–2051

Helfrich MH, Nesbitt SA, Dorey EL and Horton MA (1992) Rat osteoclasts adhere to a wide range of RGD (Arg-Gly-Asp) peptide-containing proteins, including the bone sialoproteins and fibronectin, via a beta 3 integrin. *J Bone Miner Res* **7**: 335–343

Karmatschek M, Maier I, Seibel MJ, Woitge HW, Ziegler R and Armbruster FP (1997) Improved purification of human bone sialoprotein and development of a homologous radioimmunoassay. *Clin Chem* **43**: 2076–2082

Malpas JS (1995) Clinical presentation and diagnosis. In *Myeloma Biology and Management*, Malpas JS, Bergsagel DE and Kyle RA (eds) pp. 1995. Oxford University Press: Oxford

Mundy GR and Yoneda T (1998) Bisphosphonates as anticancer drugs. *New Engl J Med* **339**: 398–400

Oldberg A, Franzen A, Heinegard D, Pierschbacher M and Ruoslahti E (1988) Identification of a bone sialoprotein receptor in osteosarcoma cells. *J Biol Chem* **263**: 19433–19436

Pecherstorfer M, Seibel MJ, Woitge HW, Horn E, Schuster J, Neuda J, Sagaster P, Köhn H, Bayer P, Thiébaud D and Ludwig H (1997) Bone resorption in multiple myeloma and in monoclonal gammopathy of undetermined significance: quantification by urinary pyridinium crosslinks of collagen. *Blood* **90**: 3743–3750

Raynal C, Delmas PD and Chenu C (1996) Bone sialoprotein stimulates in vitro bone resorption. *Endocrinology* **137**: 2347–2354

Ross FP, Chappel J, Alvarez JI, Sander D, Butler WT, Farach-Carson MC, Mintz KA, Robey PG, Teitelbaum SL and Cheresh DA (1993) Interactions between the bone matrix proteins osteopontin and bone sialoprotein and the osteoclast integrin alpha v beta 3 potentiate bone resorption. *J Biol Chem* **268**: 9901–9907

Salmon SE and Cassady JR (1993) Plasma cell neoplasms. In: *Cancer: Principles and Practice in Oncology*, vol 2, De Vita VT, Hellmann S, Rosenberg SA (eds) pp. 1984. Lippincott: Philadelphia

Seibel MJ, Woitge HW, Pecherstorfer M, Karmatschek M, Horn E, Ludwig H, Armbruster FP and Ziegler R (1996) Serum immunoreactive bone sialoprotein as a new marker of bone turnover in metabolic and malignant bone disease. *J Clin Endocrinol Metab* **81**: 3289–3294

Shapiro HS, Chen J, Wrana JL, Zhang Q, Blum M and Sodek J (1993) Characterization of porcine bone sialoprotein: primary structure and cellular expression. *Matrix* **13**: 431–440

Teoh G and Anderson KC (1997) Interaction of tumor and host cells with adhesion and extracellular matrix molecules in the development of multiple myeloma. *Hematol Oncol Clin North Am* **11**: 27–42

Uchiyama H, Barut BA and Mohrbacher AF (1993) Adhesion of human myeloma-derived cell lines to bone marrow stromal cells stimulates interleukin-6 secretion. *Blood* **82**: 3712–3720

Van Riet I and Van Camp B (1993) The involvement of adhesion molecules in the biology of multiple myeloma. *Leuk Lymphoma* **9**: 441–452

Van Riet I, Vanderkerken K, de Greef C and Van Camp B (1998) Homing behavior of the malignant cell clone in multiple myeloma. *Med Oncol* **15**: 154–164

Waltregny D, Bellahcene A, Van Riet I, Fisher LW, Young M, Fernandez P, Dewe W, deLeval J and Castronovo V (1998) Prognostic value of bone sialoprotein expression in clinically localized human prostate cancer. *J Natl Cancer Inst* **90**: 1000–1008

Woitge HW, Pecherstorfer M, Li Y, Keck A-V, Horn E, Ziegler R and Seibel MJ (1999) Novel serum markers of bone resorption: clinical assessment and comparison with established urinary indices. *J Bone Miner Res* **14**: 792–801

© 2001 Cancer Research Campaign
